# Anaemia in South American camelids – an overview of clinical and laboratory diagnostics

**DOI:** 10.1007/s11259-023-10274-z

**Published:** 2023-12-05

**Authors:** Matthias Gerhard Wagener, Hannah Marahrens, Martin Ganter

**Affiliations:** Clinic for Swine and Small Ruminants, Forensic Medicine and Ambulatory Service, University of Veterinary Medicine Hannover, Foundation, Hannover, Germany

**Keywords:** Alpaca, Llama, South American camelids, Haematology, Anaemia, Regenerative, FAMACHA© score, BCS, Body Condition score

## Abstract

South American camelids (SACs) play an increasing role in veterinary care in Europe. Many alpacas or llamas presented to veterinarians suffer from anaemia, regularly with a packed cell volume (PCV) below 0.10 l/l, which is a life-threatening condition for the animals. This review article presents clinical and laboratory diagnostic tools for the diagnosis of anaemia in SACs. Clinical identification of anaemic animals can be performed by assessing the FAMACHA© score and the Body Condition Score (BCS), since anaemia in alpacas and llamas correlates with pale mucous membranes and a lowered BCS. Haematological examination of a blood sample can provide a more differentiated diagnosis of anaemia in SACs. A common finding is regenerative anaemia with an increased number of reticulocytes that is often caused by blood loss due to *Haemonchus contortus*. Changes in a blood smear from an alpaca or llama with regenerative anaemia may include normoblasts (nucleated red blood cells), anisocytosis, poikilocytosis, polychromasia, Howell-Jolly bodies or basophilic stippling. Furthermore, non-regenerative anaemia, often caused by trace element deficiency or cachexia, can also occur.

## Introduction

Alpacas and llamas are increasingly kept outside zoos on farms or by private individuals in Europe (Davis et al. 1998; Hengrave Burri et al. 2005a; Neubert et al. 2021). The veterinary care of these South American camelids (SACs) is therefore also of increasing importance (Neubert et al. 2021; Wagner et al. 2022a, b). Retrospective and epidemiological studies on health problems in SACs from different countries show that alpacas and llamas frequently suffer from diseases of the gastrointestinal tract, primarily endoparasitoses or gastric ulcers, next to diseases of the skin, respiratory tract, liver or teeth (Björklund 2014; Clarke and Breuer 2022; D’Alterio et al. 2005; Neubert 2022; Neubert et al. 2022; Niehaus and Anderson 2007; Shapiro et al. 2005; Theuß et al. 2014; Twomey et al. 2014). Many alpacas and llamas presented for veterinary examination also exhibit anaemia (Wagener et al. 2021). Anaemia in veterinary medicine is usually diagnosed by a decrease in red blood cell (RBC) count, haemoglobin content or packed cell volume (PCV) (Moritz 2014). These parameters can be used to calculate erythrocyte indices, which further help to characterise anaemia. These include mean corpuscular volume (MCV), mean corpuscular haemoglobin (MCH) and mean corpuscular haemoglobin concentration (MCHC) (Sarma 1990). According to Wittek and Franz, anaemia in SACs can be classified into different degrees of severity (Wittek and Franz 2021): PCV: 0.25 − 0.20 l/l: mild anaemia; PCV: 0.20 − 0.15 l/l: moderate anaemia; PCV: 0.15 − 0.10 l/l: severe anaemia; PCV < 0.10 l/l: fatal anaemia.

In a retrospective study of 300 SACs presented to our clinic, comparison with the reference values of Hengrave Burri et al. (2005b) revealed that 49% of llamas and 55% of alpacas had PCV levels that were below the reference interval (Wagener et al. 2021). Some of these animals were found to have severe or fatal anaemia with a PCV of less than 0.10 l/l, with the lowest recorded values being 0.04 l/l in both llamas and alpacas (Wagener et al. 2021).

This review paper will explain causes and diagnosis of anaemia in alpacas and lamas. On the one hand, the FAMACHA© score, adapted from small ruminants, will be presented as a clinical tool; on the other hand, different laboratory parameters will be discussed.

## Blood sampling in South American camelids

For haematological diagnostics, it is recommended to utilize an EDTA blood sample (Milne and Scott 2006; Otter 2013). If other parameters such as metabolites, enzyme activities, minerals or trace elements are to be examined, heparin or serum tubes should also be used (Otter 2013). Venous blood samples can be taken from the jugular vein in SACs (Foster et al. 2009), but it should be considered that the jugular vein and the carotid artery are in close contact (Gauly et al. 1998). Particular care should be taken to avoid clotting when taking blood samples for haematology, as this can lead to incorrect results, especially when employing haematological analysers (Humann-Ziehank and Ganter 2012).

## Classification of anaemia

Anaemia in alpacas and llamas have different causes and can be regenerative or non-regenerative. The distinction is based on the erythropoietic response to the anaemia. If the erythropoietic response is insufficient, the anaemia is non-regenerative (Tyler and Cowell 1996).

## Regenerative anaemia

Regenerative anaemia in SACs may be caused by either blood loss or haemolysis (Tvedten 2022). In alpacas and llamas, severe or fatal anaemia due to blood loss caused by infection with *Haemonchus contortus* occurs regularly, especially in summer and autumn (Edwards et al. 2016; Franz et al. 2015). The female adult nematodes attach the well-vascularised mucosa of the third compartment of the host animal to feed on the blood of the host (Arsenopoulos et al. 2021; Hoogmoed et al. 2003). Since on average approximately 50 μL of blood is lost daily per female worm in the third compartment, severe blood loss can occur rapidly (Clark et al. 1962). In the case of 1000 bloodsucking nematodes in the third compartment, the daily blood loss would be 50 mL, which usually cannot be compensated and leads to a significant decrease in the PCV (Arsenopoulos et al. 2021). Several studies have shown that gastrointestinal nematodes, which include *H. contortus*, have a high prevalence among SACs worldwide (Gillespie et al. 2010; Kultscher et al. 2018; Muhm 2021; Rashid et al. 2019). This nematode is also regularly detected in the third compartment of SACs during post-mortem examinations (Björklund 2014; Edwards et al. 2016; Theuß et al. 2014). According to Kultscher et al. (2018), the majority of deaths in alpacas caused by endoparasites can be attributed to haemonchosis.

Alpacas and llamas with haemonchosis can have very low PCV, which can decrease to 0.05 l/l (Frahm et al. 2016; Wagener et al. 2018a, 2020). Although this is a life-threatening condition for the affected animals due to insufficient oxygen transport, intensive therapy, including the administration of blood transfusions, can be successful (Frahm et al. 2016; Wagener et al. 2018a).

*Haemonchus contortus* probably plays the most significant role for anaemia caused by blood loss in SACs, but blood loss can also occur as a result of injuries due to fractures, rank fighting among stallions or surgical procedures (Newman and Anderson 2009; Tibary et al. 2008; Tvedten 2022).

In other species, blood loss also frequently occurs in association with gastric ulcers (Braun et al. 2019; Friendship 2004; Kavanagh 1994; Sullivan and Yool 1998). Damage to the gastric mucosa can result in massive intraluminal bleeding, which can be detected by faecal occult blood tests. In SACs, gastric ulcers are a common problem, but they do not lead to anaemia in every case, and an occult blood test does not provide a reliable result (Neubert et al. 2022; Smith et al. 1994; Whitehead 2013).

**Fig. 1:**
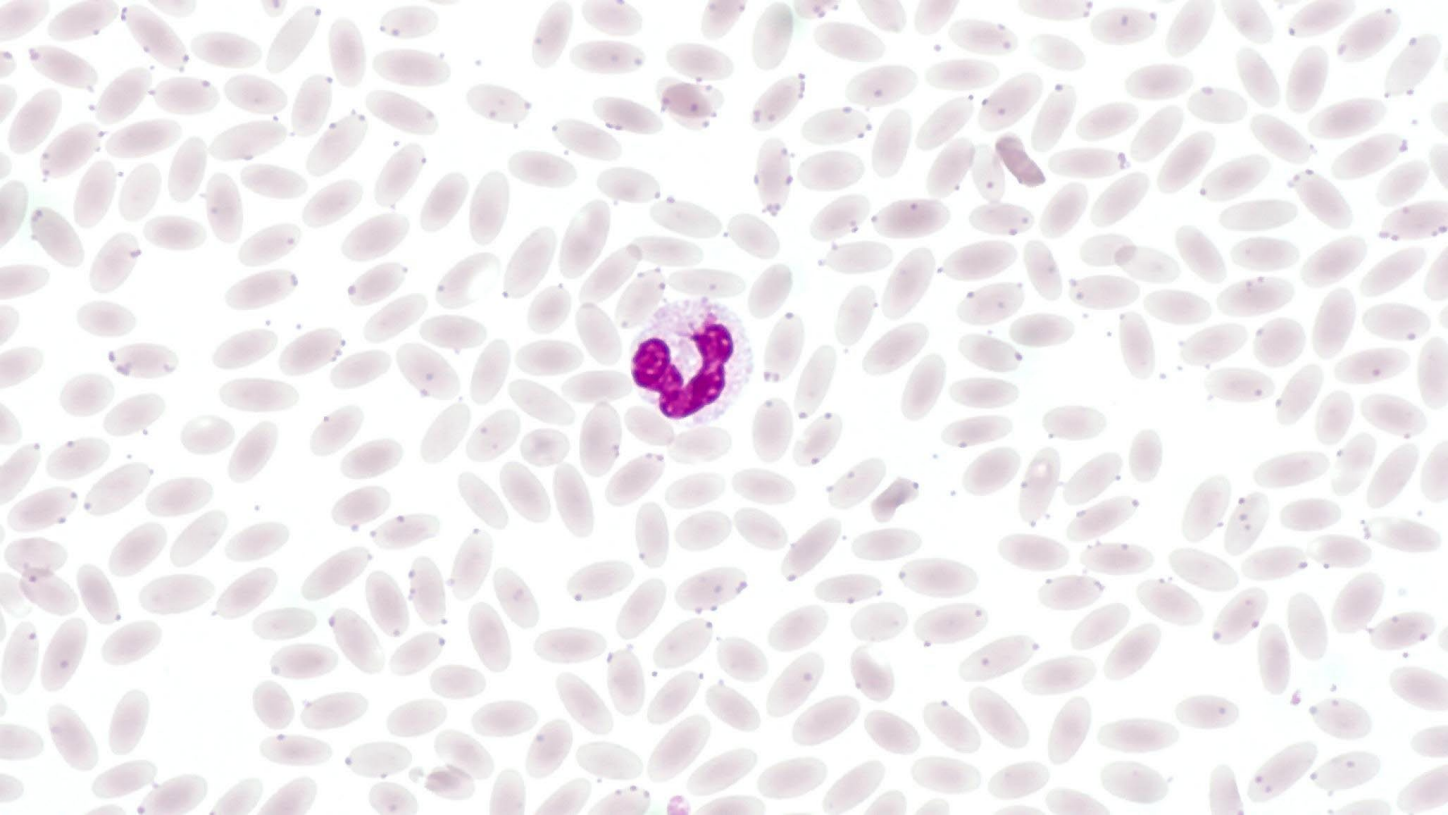
Blood smear from an alpaca infected with *Candidatus* Mycoplasma haemolamae. The haemotrophic mycoplasmas appear as basophilic dots on the erythrocytes. However, infection should be confirmed by PCR as microscopic examination is less sensitive and mycoplasmas are not visible in every blood smear. The leukocyte located in the centre is a band neutrophil. Pappenheim stain

Another pathogen associated with regenerative anaemia in alpacas and llamas is *Candidatus* Mycoplasma haemolamae (Tornquist 2009), a haemotrophic bacteria that infects the surface of erythrocytes and is regularly found in SACs (Fig. 1) (Crosse et al. 2012; Kaufmann et al. 2011; Tornquist 2009; Vap and Bohn 2015). Haemotrophic pathogens can lead to the increased premature destruction of erythrocytes, causing haemolytic anaemia (Messick 2004; Paul et al. 2020). However, in healthy alpacas and llamas, this pathogen is not necessarily associated with changes of the RBC count (Viesselmann et al. 2019b), according to Crosse et al. (2012), clinical signs are likely to occur only as a result of stress or co-morbidities. For other haematotrophic pathogens such as *Babesia* spp., *Anaplasma* spp. or *Theileria* spp. that play an important role in small ruminants (Stuen 2020), there are no detailed descriptions in the scientific literature concerning SACs. Nonetheless, haemolysis has been described in alpacas after ingestion of red maple leaves (DeWitt et al. 2004). Furthermore, Peauroi et al. described the case of an alpaca suffering from haematuria and anaemia due to bracken fern intoxication (Peauroi et al. 1995).

Water intoxication with subsequent haemolysis, that has been described in cattle (Gilchrist 1996), does not play a role in camelids. The erythrocytes of camelids have a very high osmotic resistance due to their special elliptical morphology, which enables the animals to absorb large amounts of fluid at once (Livne and Kuiper 1973; Schmidt-Nielsen et al. 1956; Smith et al. 1979). Vap and Bohn (2015) also mention idiopathic immune-related haemolytic anaemia (IMHA), which is a regular occurrence in other species (Kohn et al. 2006; Nassiri et al. 2011; Reimer et al. 1999; Wilkerson et al. 2000), as a differential diagnosis to anaemia in a review of haematology in SACs. However, there are no specific descriptions of this condition in alpacas or llamas in the literature yet.

## Haematological features of regenerative anaemia

**Fig. 2:**
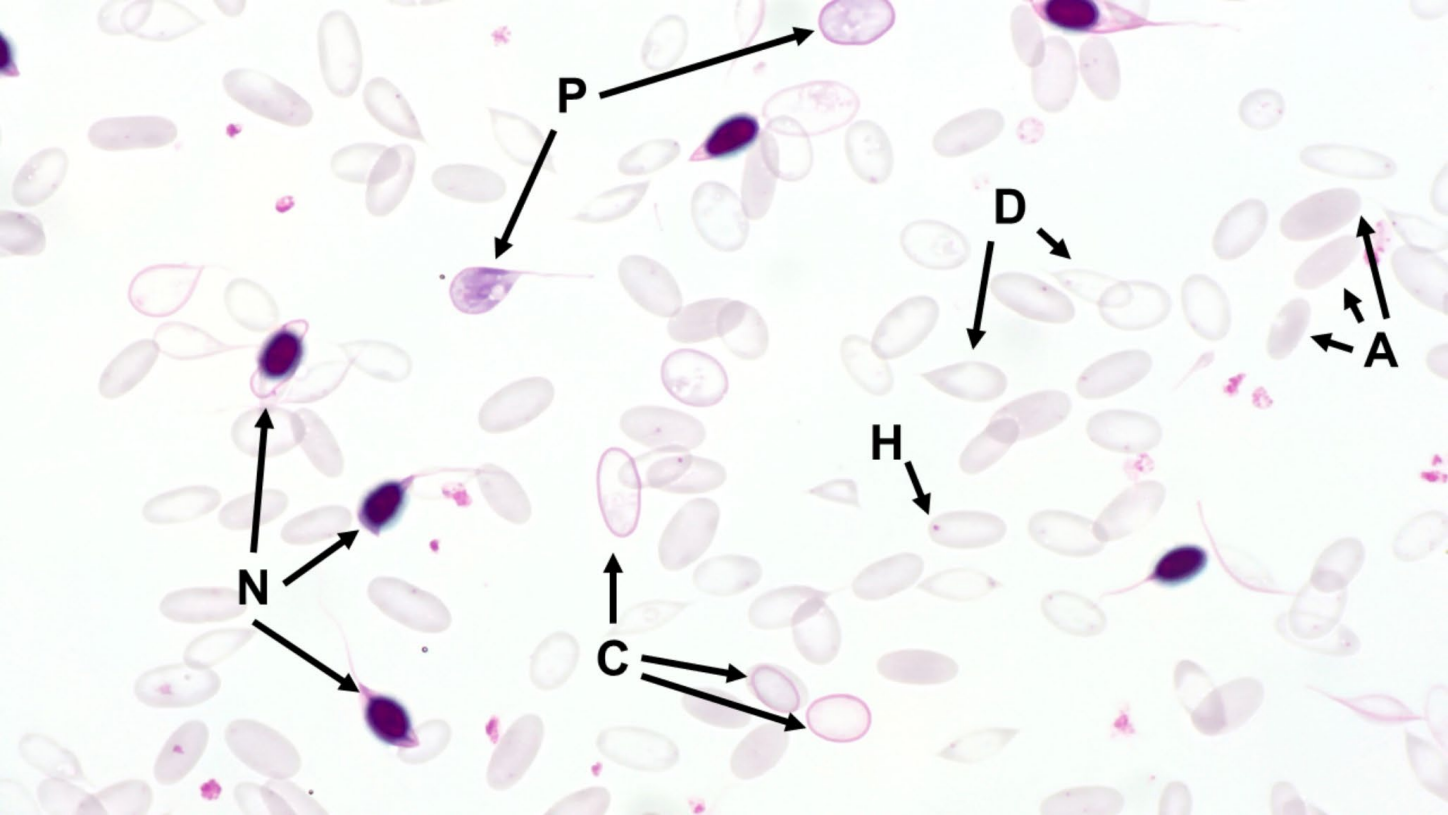
Blood smear of an alpaca with severe anaemia (PCV: 0.05 l/l) due to haemonchosis. The blood smear of the animal reveals several characteristics that are common in anaemic SACs. The cells are widely spaced throughout the blood smear. A: Anisocytosis (variation in size of the cells); C: Cabot rings (thread-like structures that either represent a prominent border around the erythrocytes, or have the shape of the figure eight); D: Dacrocytes (teardrop-shaped erythrocytes); H: Howell-Jolly bodies (nuclear remnants that can vary in size and appear as single, round, basophilic dots in erythrocytes); N: Normoblasts (nucleated red blood cells); P: Polychromasia (reticulocytes that are stained blue due to the higher RNA-content). Pappenheim stain

**Fig. 3:**
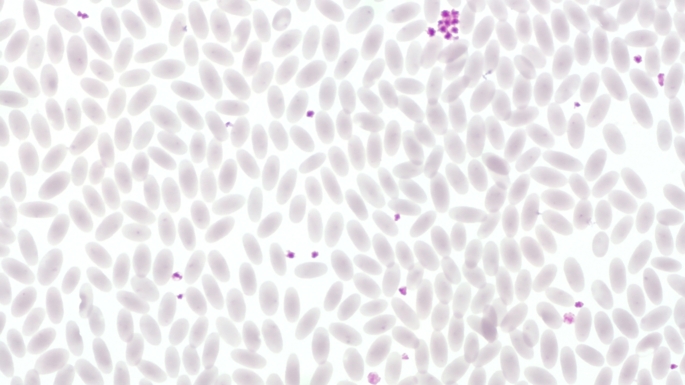
Blood smear of an alpaca without anaemia with physiological erythrocyte morphology. Pappenheim stain

In regenerative anaemia, haematological findings may include increased numbers of reticulocytes, normoblasts as well as the appearance of anisocytosis, polychromasia, poikilocytosis, Howell-Jolly bodies or basophilic stippling (Fig. 2) (Katsogiannou et al. 2018; Knottenbelt 2001; Otter 2013; Tasker 2012; Tvedten 2022; Tyler and Cowell 1996). In animals with a physiological PCV, on the other hand, the erythrocytes usually look uniform (Fig. 3). However, species-specific differences should be taken into account. In horses, reticulocytes are rather rare even in cases of anaemia (Barrelet and Ricketts 2002; Lording 2008); in cats, Howell-Jolly bodies occur regularly even under physiological circumstances (Harvey 2017; Papasouliotis and Murphy 2021).

## Reticulocytes

**Fig. 4:**
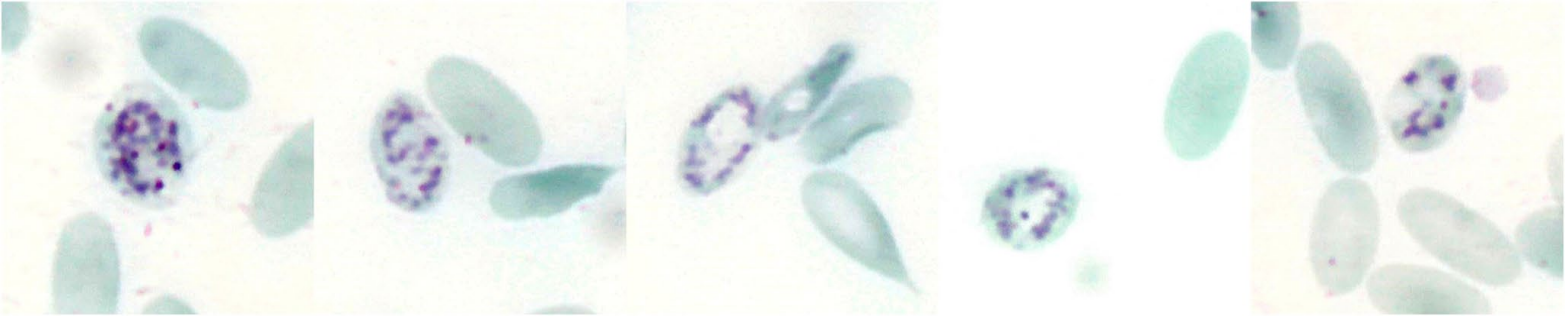
Reticulocytes. Reticulocytes stained with brilliant cresyl blue showing a reticular structure, the *Substantia granulofilamentosa*. Polychromatic erythrocytes in the Pappenheim stain are also reticulocytes, but not every reticulocyte is polychromatic. For microscopic quantification, supravital staining with brilliant cresyl blue or new methylene blue should therefore be performed. Brilliant cresyl blue stain

**Fig. 5:**
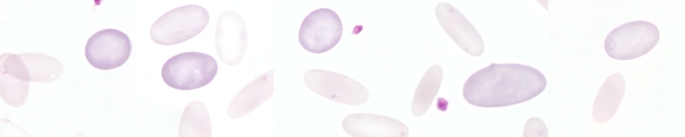
Polychromasia in blood smears from anaemic alpacas. Polychromatic erythrocytes showing a bluish staining in Romanowsky stains are reticulocytes. Pappenheim stain

A simple quantifiable parameter that provides information about haematopoietic activity is the reticulocyte count (Fig. 4). Reticulocytes are juvenile erythrocytes that are usually larger than the mature RBC which comprise the majority of the circulating RBC count (Tornquist 2009; Vap and Bohn 2015; Wagener et al. 2018b). Reticulocytes appear as polychromatophilic in Romanowsky stains (Fig. 5). For microscopic quantification, supravital stains such as new methylene blue or brilliant cresyl blue stain can be used (Papasouliotis and Murphy 2021; Vap and Bohn 2015; Wagener et al. 2018b) (Fig. 4). These stains form precipitates with the ribonucleic acid, that is still present in high concentrations in the young cells and which appears as a stained reticular structure in light microscopy, the *Substantia granulofilamentosa* (Fig. 4) (Heimpel et al. 2010; Papasouliotis and Murphy 2021). Although reticulocytes can also be determined using automated methods, it is not yet known how accurate these examinations are for reticulocytes in camelids.

## Normoblasts (nucleated RBC)

**Fig. 6:**
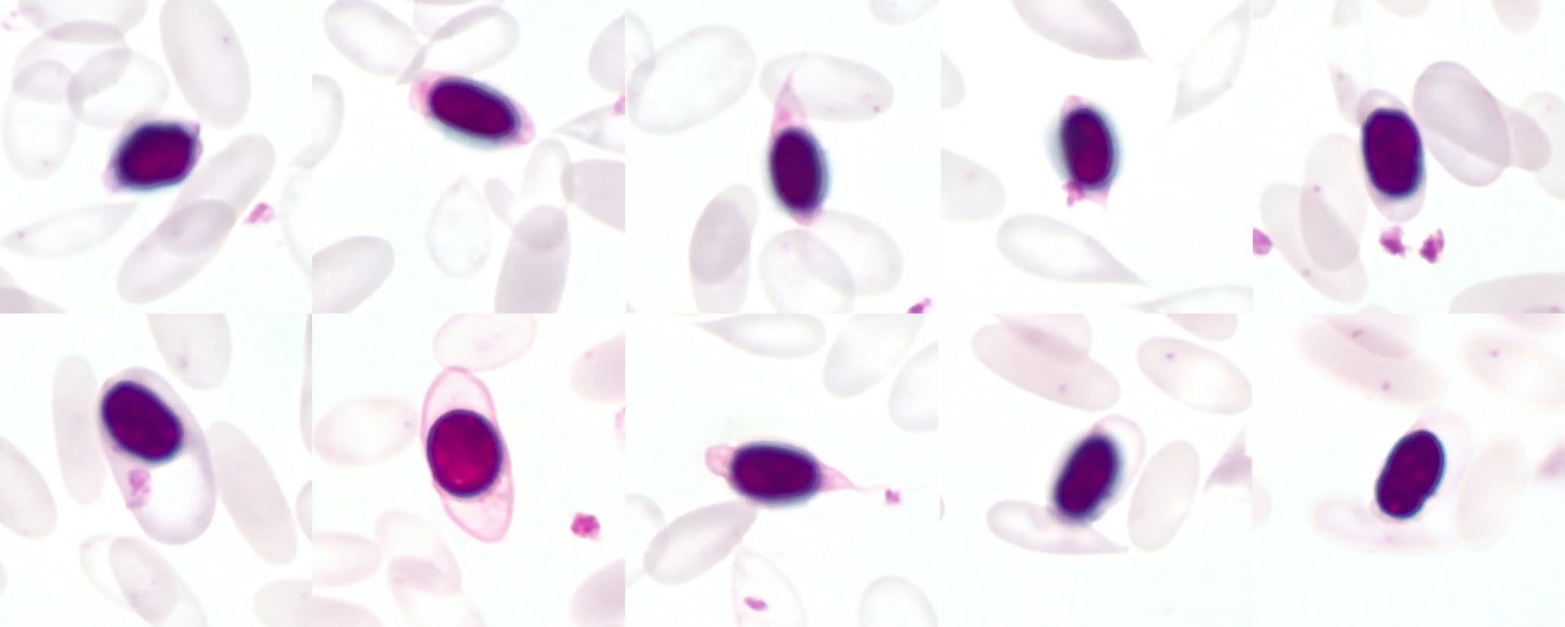
Normoblasts (nucleated red blood cells) in alpacas with anaemia. The nuclei of normoblasts typically do not lie exactly in the same plane of focus as erythrocytes. This can be seen in the two image sections at the bottom right. Both times, the cell is the same, but the focus was chosen differently. Pappenheim stain

Normoblasts are nucleated RBCs (Fig. 6) that represent immature erythroid cells from the bone marrow or in case of extramedullary haematopoiesis from the liver or spleen (Canfield 1998; Wagener et al. 2018b). According to Ciesla (2007), there are six different stages in the maturation of RBCs. Depending on the nomenclature, these are called Pronormoblast (Rubriblast); Basophilic normoblast (Prorubricyte); Polychromatophilic normoblast (Rubricyte); Orthochromatic normoblast (Metarubricyte); Reticulocyte; Erythrocyte. Whereas the cytoplasm of pronormoblasts is dark marine blue, the cytoplasm of orthochromatic normoblasts is pale blue with orange-red colour tinges (Ciesla 2007).

An increased occurrence in the peripheral blood can therefore be an indication of regenerative anaemia (Jones and Allison 2007; Otter 2013; Tyler and Cowell 1996). However, the presence of normoblasts in the peripheral blood can also be associated with many other diseases like onco-haematological conditions (Danise et al. 2012; Stachon et al. 2002). In contrast to other species, in which normoblasts are found only very rarely in the peripheral blood, they are found regularly in healthy SACs (Dawson et al. 2011; Fowler and Zinkl 1989) and are quite common in anaemic alpacas and llamas (Morin et al. 1993; Wagener et al. 2018a, 2020, 2021) (Figs. 2 and 6).

## Reference values for reticulocytes and normoblasts

The reference values for reticulocytes and normoblasts for SACs given in the literature vary significantly. Depending on age and sex, Al-Izzi et al. (2004) give 0–5 normoblasts per 100 leukocytes and 0-0.8% reticulocytes (as a percentage of erythrocytes) as reference values for llamas. According to Fowler and Zinkl (1989), 0–3 normoblasts per 100 leukocytes and 0-0.5% reticulocytes are considered physiological in adult llamas, up to 26 normoblasts per 100 leukocytes and up to 7.5% reticulocytes are given as normal values for newborn crias. Van Houten et al. (1992) chose a different form of indication; in their study, the reference value for reticulocytes in llamas is given as 12,000 to 79,000/μL (corresponding to 12–79 G/l). For adult alpacas, Dawson et al. (2011) report 0–3 normoblasts per 100 leukocytes. Reference values for reticulocytes in alpacas as well as a classification into different degrees of regenerative capacity based on reticulocyte count, as reported for other species (Knottenbelt 2001; Tasker 2012), are currently not available for SACs. Knottenbelt’s data for dogs could serve as orientation, according to which > 1% reticulocytes (of 1000 erythrocytes) would indicate weak regeneration and > 20% reticulocytes strong regeneration (Knottenbelt 2001).

## Erythrocyte morphology in anaemia

Blood smears from alpacas and llamas with severe or fatal anaemia usually show several changes in erythrocyte morphology, which can be determined semi-quantitatively in the monolayer of the blood smear (Fig. 2) (Wagener et al. 2018b). Changes of the erythrocytes in anaemic conditions can generally be addressed to anisocytosis (variation in size of the cells), poikilocytosis (variation in shape of the cells) and polychromasia (presence of larger cells with a blue tint) (Hawkins and Hill 2014). Polychromasia (Fig. 5) is a result of an increased number of reticulocytes, that are stained blue due to the higher RNA content (Bain 2005; Hawkins and Hill 2014). Poikilocytosis can further be subdivided based on characteristic erythrocyte shapes. In SACs, dacrocytes (teardrop-shaped erythrocytes) are frequently present; their increased occurrence could provide evidence of iron deficiency (Morin et al. 1992; Murray et al. 2001; Wernery et al. 1999) (Fig. 2).

## Inclusion bodies in erythrocytes

**Fig. 7:**
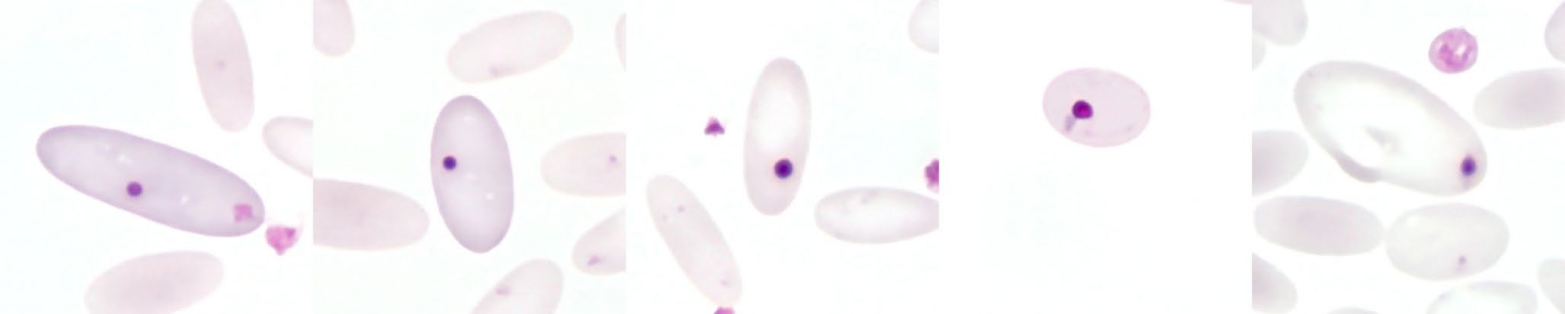
Howell-Jolly bodies. These are nuclear remnants that can vary in size and appear as single, round, basophilic dots in erythrocytes. Pappenheim stain

Howell-Jolly bodies are nuclear remnants that can vary in size and appear as single, round, basophilic dots in erythrocytes in blood smears after Romanowsky staining (Fig. 7) (Barger 2022; Jones and Allison 2007; Wernery et al. 1999). According to Azwai et al. they can be found in 6% of the red blood cells of adult healthy llamas (Azwai et al. 2007). In contrast, erythrocytes with basophilic stippling have multiple small basophilic spots (Jones and Allison 2007) consisting of aggregates of ribosomes and polyribosomes (Harvey 2017). The nuclear remnants that form the Howell-Jolly bodies can be easily confused with haemotrophic mycoplasmas (Fig. 1) because of their morphology (Sykes 2010).

If an infection with *Candidatus* M. haemolamae is suspected, a PCR should be performed for confirmation (Meli et al. 2010; Tornquist et al. 2009), as microscopic examination (Fig. 1) alone has only low sensitivity compared with PCR (Dittmer et al. 2017; Kaufmann et al. 2009). Moreover, it is necessary to examine fresh blood smears (Tornquist 2009; Whitehead 2013), which is probably not ensured in every case, for example, when samples are taken at the weekend and processed only after a time delay. The pathogen is also widespread in asymptomatic SACs (Franz et al. 2016; Kaufmann et al. 2009, 2011; Ramos et al. 2021). In healthy alpacas and llamas, an infection with *Candidatus* M. haemolamae is not necessarily associated with anaemia (Viesselmann et al. 2019b).

Besides mycoplasmas, other pathogens that are not primary related to anaemia such as *Anaplasma phagocytophilum* or different protozoa may be present in the blood smears of SACs that are infected with these pathogens (Lascola et al. 2009; Wernery et al. 1999). The examination of blood smears is important, as blood parasites often stay undetected when the blood sample is examined with an analyser only.

## Cabot rings

**Fig. 8:**
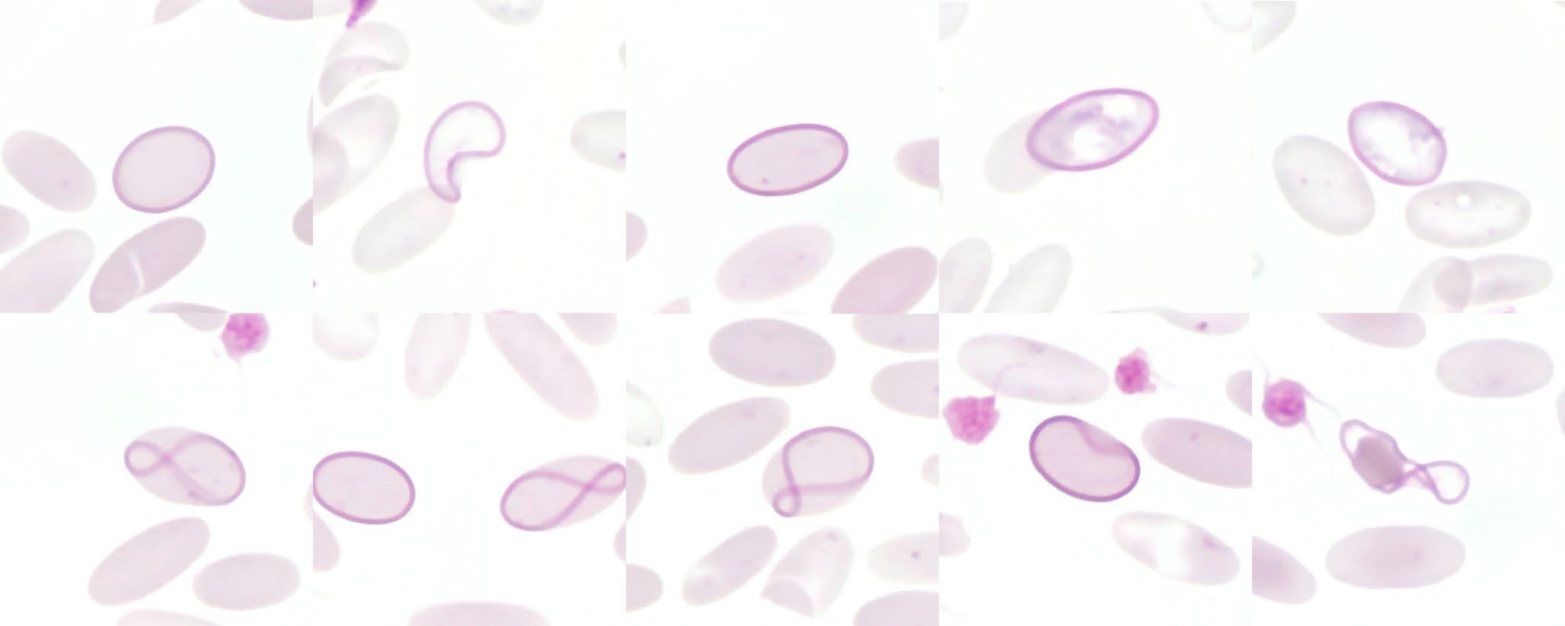
Cabot rings in blood smears from alpacas. Cabot rings occur regularly even in healthy SACs and can either represent a border around the cell or be shaped like the figure eight. To date, little is known about the role of Cabot rings in SACs. Pappenheim stain

Cabot rings are thread-like structures that either represent a prominent border around the erythrocytes or have the shape of a figure eight (Fig. 8) (Tornquist 2009). Little is known to date about the role of Cabot rings in SACs. They may be denatured membrane proteins or a combination of histones and non-haemoglobin iron (Tornquist 2009). They also occur regularly in healthy SACs and can be seen particularly well in the brilliant cresyl blue stain (Azwai et al. 2007; Hawkey and Gulland 1988). Azwai et al. (2007) found Cabot rings in 1–5% of erythrocytes in each animal when they examined 10 healthy adult male and female llamas (Fig. 8).

## Problems with automated measurement of anaemic blood samples

Automated measurement of blood samples from anaemic alpacas or llamas have to be interpreted carefully, as they might lead to wrong results. Especially normoblasts, that may be present in the peripheral blood, might be misclassified as leukocytes during automated measurement (Jones and Allison 2007; Pinches 2006a). Newer haematological analysers are able to detect normoblasts to some extent, but there are still sources of error (Briggs 2009; Da Rin et al. 2017). Therefore, a microscopic examination of a blood smear is necessary in SACs with anaemia, as normoblasts could be found in the blood smears of one-third of the alpacas and llamas presented to the clinic and animals with a lower PCV usually had a higher normoblast count (Wagener et al. 2021). Since misclassification of normoblasts as leukocytes leads to mistakes in the white blood cell (WBC) count (Wagener et al. 2020), a mathematical correction should be calculated if normoblasts are present (Wagener et al. 2018b). The formula for this is:

*True WBC (G/l) = counted nucleated cells (G/l) x 100 / (100 + number of normoblasts per 100 leukocytes)*.

Another parameter that is regularly determined by haematological counters working with the conductometric principle is the “red cell distribution width” (RDW) (Evans and Jehle 1991). The RDW provides a quantitative measurement of the anisocytosis of the cells (Evans and Jehle 1991). In regenerative anaemia, which is associated with a higher proportion of reticulocytes, the RDW increases (Roberts and EL Badawi 1985). Studies in dogs have shown that RDW and MCV can be used to distinguish regenerative from non-regenerative anaemia in this species (Neiger et al. 2002). However, little is known about RDW in SACs to date. Juvenile alpacas have significantly higher RDW´s than adults (Husakova et al. 2015); which, as well as the higher number of reticulocytes in juvenile SACs (Fowler and Zinkl 1989), could be explained by higher haematopoietic activity. Whether RDW is also suitable in SACs with severe or fatal anaemia to distinguish regenerative from non-regenerative anaemia remains questionable. Therefore, the decision whether anaemia in an alpaca or llama is regenerative or non-regenerative should be made by the quantifiable number of reticulocytes.

Viesselmann et al. (2019a) studied RBC parameters in 25 SACs in an analyser (ADVIA 2120), they observed significant deviations in MCV and MCHC from the reference method in two animals with fatal anaemia (PCV: 0.07 l/l and 0.08 l/l). Vap and Bohn (2015) also report falsely elevated MCHC in the automated measurement of blood samples from SACs and attribute this to the fact that in some cases too few erythrocytes have been collected. Due to the elliptical erythrocyte morphology, discrepancies may also occur between PCV, that is determined by centrifugation in a microhematocrit capillary and the automated measurement of the haematocrit (HCT) in an analyser, as HCT is calculated mathematically from MCV and the RBC count (Jones and Allison 2007; Weiser et al. 1992). Therefore, it is necessary to detect the PCV by centrifugation in a microhematocrit capillary in anaemic SACs (Wagener et al. 2018b). A microscopic RBC count should also be considered if the MCHC is significantly elevated.

Nevertheless, some haematological parameters may also be influenced by preanalytical factors, for example, as a result of drug treatments or excessively long storage of EDTA blood samples (Pinches 2006b; Tvedten 2022).

## Investigation of bone marrow

Investigation of the haematopoietic activity of the bone marrow can also be performed in living animals by bone marrow aspiration at the sternum (Higgs 2019; Steinberg et al. 2008). Only little information about bone marrow cytology of SACs is available so far. In bone marrow aspirates from seven healthy llamas, Andreasen et al. found between 50% and 75% overall marrow cellularity, with a ratio of myeloid to nucleated erythropoietic progenitor cells varying between 0.9 and 2.9 (Andreasen et al. 1994). However, in their natural habitat at high altitudes, SACs also reveal much higher proportions of erythropoietic progenitor cells in the bone marrow (Reynafarje et al. 1968). Examination of a bone marrow punctate is not necessary in regenerative anaemia but may be useful in prolonged non-regenerative anaemia; other indications can be persistent leucopaenia or persistent thrombocytopaenia (Higgs 2019; Tornquist 2009).

## Non-regenerative anaemia

If there is a depletion of erythropoietic progenitor cells in the bone marrow, or if there are no or very few reticulocytes found in the peripheral blood, the anaemia has a non-regenerative character. According to scientific literature, non-regenerative anaemia in alpacas and llamas can be caused by copper, cobalt or iron deficiency (Andrews and Cox 1997; Foster et al. 2009; Morin et al. 1992). In the case of non-regenerative anaemia, erythrocyte indices can give hints to possible causes (Tvedten 2022). In llamas with iron deficiency anaemia, Morin et al. (1993) observed a decrease in MCV and MCHC. However, no descriptions concerning the changes in erythrocyte indices in SACs with cobalt or copper deficiency could be found in the current literature. In ruminants, cobalt deficiency is associated with macrocytic anaemia (increased MCV), whereas copper deficiency can lead to microcytic anaemia (decreased MCV) (Roland et al. 2014). A slightly lower MCHC may indicate regeneration (Jones and Allison 2007).

Other causes of non-regenerative anaemia include chronic inflammation as well as bone marrow diseases (Tornquist 2009). Anaemia associated with acute myeloid leukaemia has been reported in alpacas before: Steinberg et al. (2008) described the case of an 18-year-old female alpaca with non-regenerative, macrocytic, hypochromic anaemia (PCV: 0.20 l/l) associated with acute myeloid leukaemia, Murray et al. (2001) described the case of a pregnant two-year-old alpaca with normocytic anaemia (PCV: 0.10 l/l) that changed to macrocytic anaemia.

Many alpacas and llamas presented to the clinic suffered from emaciation or cachexia on the subsequent pathological examination (Neubert 2022; Neubert et al. 2022). In cachectic animals, the haematopoietic activity of the bone marrow may be reduced, resulting in non-regenerative anaemia (Pointer et al. 2013; Travlos 2006). Another cause of non-regenerative anaemia may be erythropoietin deficiency (Grimes and Fry 2015), which could play a role as a consequence of kidney disease especially in cats (Chalhoub et al. 2011). However, there are no more concrete data on this condition in SACs to date (Tornquist 2009).

The distinction between regenerative and non-regenerative anaemia in alpacas and llamas is not always clear, as the above-mentioned characteristics of regeneration are not observed there in every case (Tornquist 2009). Furthermore, a strict separation into regenerative and non-regenerative anaemia cannot be made in practice for every case. For example, in the case of haemonchosis, the loss of blood can also lead to iron deficiency, which may delay regeneration (Wagener et al. 2018a).

## Haematological reference values

Male and female SACs are both equally affected by anaemia (Wagener et al. 2021). However, female alpacas and llamas have significantly lower RBC counts and haemoglobin levels than males, even under physiological conditions (Hengrave Burri et al. 2005b; Husakova et al. 2015). For the evaluation of haematological findings in SACs, a number of published reference values can be found in the scientific literature. There are specific reference values for alpaca and llamas as well as for different age groups (Dawson et al. 2011; Fowler and Zinkl 1989; Hajduk 1992; Hengrave Burri et al. 2005b; Husakova et al. 2015; Stanitznig et al. 2018). However, when using reference values, it should be ensured that the method that was used for establishing the reference value is consistent with the method used in the laboratory.

## Clinical diagnosis of anaemia

While anaemia is a common finding in alpacas and llamas, it is not necessarily apparent to the animal at first glance, as SACs could compensate for anaemia for a long time. Therefore, it is important to identify alpacas and llamas at risk of anaemia in time. Suitable tools are the FAMACHA© score and the Body Condition Score (BCS) (Storey et al. 2017; Wagener et al. 2022; Wagener and Ganter 2020).

## The FAMACHA© score

A decrease in the haemoglobin content of the blood causes the mucous membranes to appear paler, which is used in the assessment of the FAMACHA© score. This scoring system was developed in South Africa in the 1990s for targeted selective treatment of small ruminants suffering from haemonchosis (Bath et al. 1996; Van Wyk and Bath 2002). The term “FAMACHA” is an acronym for an image panel developed according to an idea by Dr. Francois “Faffa” Malan (FAffa Malan CHArt) (Bath et al. 1996; Van Wyk and Bath 2002). It contains five different shades of red, which are assigned to a score from 1 to 5. The FAMACHA© card is held next to the eye of the animal for evaluation, thus allowing a direct comparison with the colour of the conjunctival mucous membranes (Bath et al. 1996; Maia et al. 2014). Score 1 corresponds to a physiological red colouration of the conjunctiva, score 5 to almost white conjunctiva. The FAMACHA© score is established worldwide in the herd management of small ruminants (Naeem et al. 2021; Sabatini et al. 2023).

Besides small ruminants, the FAMACHA© score has also been applied to alpacas and llamas (Cocquyt et al. 2016; Galvan et al. 2012; Storey et al. 2017; Viesselmann et al. 2019b; Wagener et al. 2021, 2022). Storey et al. (2017) investigated the usefulness of the FAMACHA© score in 859 SACs. They concluded that using FAMACHA© scores ≥ 3 and a PCV ≤ 0.15 l/l indicating anaemia had the best sensitivity of 96.4%, whereas the best specificity (94.2%) was achieved with FAMACHA© scores ≥ 4 and a PCV ≤ 0.20 l/l (Storey et al. 2017). In those SACs presented to the veterinary clinic, there were also significant correlations between the FAMACHA© score and PCV; animals with a higher FAMACHA© score had a lower PCV (Wagener et al. 2021). SACs with a FAMACHA© score of 5 had a mean PCV of 0.06 l/l (Wagener et al. 2022).

For the practical examination, appropriate lighting conditions, preferably daylight, should be ensured; the examining person should stand sideways next to the animal (Williamson 2014). For presentation of the conjunctives, the lower eyelid should be gently pulled down while the globe is pushed into the orbit by applying light pressure on the upper eyelid (Lopez 2021; Wagener et al. 2022). Since redness may cause the FAMACHA© score to be falsely underestimated, both eyes of the animal should be examined and the paler side used for assessment (Lopez 2021).

According to Lopez (Lopez 2021), different interventions should be taken in SACs depending on the score:

- Scores 1 and 2: no treatment is necessary unless signs of clinical illness like weight loss or diarrhoea are present.
- Score 3: more frequent monitoring is required. Treatment, especially deworming, is necessary if the animal is pregnant, if more than 10 per cent of the flock has a score of > 3 or if the nutritional status is poor.
- Scores 4 and 5: deworming is always required.

Although the FAMACHA© score is a simple clinical tool to identify anaemic animals, it does not provide a diagnosis on the cause of the anaemia. Thus, further diagnostics such as faecal egg count are required (Koopmann et al. 2006; Wagener et al. 2022).

## Relationship of nutritional status and anaemia

Another clinical parameter closely associated with anaemia is the Body Condition Score (BCS). Studies by Storey et al. (Storey et al. 2017) as well as the analysis of data from our clinic (Wagener et al. 2021) showed significant correlations of BCS and PCVs in SACs. Alpacas and llamas with poor nutritional status are more likely to suffer from anaemia than alpacas and llamas with an adequate BCS (Wagener et al. 2021). A relationship between decreased BCS and anaemia is also documented for species such as horses or sheep (Munoz et al. 2010; Torres-Chable et al. 2020). Therefore, the regular assessment of the BCS represents an important and reliable clinical tool which should be combined with the assessment of the FAMACHA© score in lean animals (Wagener et al. 2022, 2023).

## Treatment of anaemia

Treatment depends on the cause of anaemia in each individual case. In the case of haemonchosis, a suitable anthelmintic should be administered, but possible anthelmintic resistance that may be present should be considered (Galvan et al. 2012; Jabbar et al. 2013). A review of endoparasites in SACs and their pharmacotherapeutic control was previously published by Franz et al. (2015). Trace element deficiencies should be adjusted by supplementation, but excessive doses that can lead to poisoning should be avoided (Carmalt et al. 2001; Junge and Thornburg 1989). A review of nutritional requirements in SAC was previously published by Van Saun (2006).

## Blood transfusion

A fatal PCV of < 0.10 l/l usually requires a blood transfusion, since it is a life-threatening condition for the animal (Wittek and Franz 2021). Blood transfusions have been previously performed in SACs due to different conditions (DeWitt et al. 2004; Frahm et al. 2016; Galvan et al. 2012; Luethy et al. 2017; Wagener et al. 2018a). However, depending on the author, recommendations for blood transfusion vary between a PCV of < 0.08 l/l (Tibary et al. 2008) and < 0.15 l/l (Fowler and Bravo 2010). To compensate for the lack of erythrocytes, whole blood from a healthy donor animal can be transfused into the anaemic animal. From a healthy llama or alpaca, 20% of the circulating blood volume or 1.6% of the body weight can be taken for transfusion (Credille and Epstein 2016). Whitehead (2013) suggests transfusion of a single unit of 450 mL of blood from a healthy donor with a PCV between 0.24 and 0.28 l/l.

The following formula can be used for calculating the transfusion volume (Luethy et al. 2017):

*Transfusion volume (L) = volume of distribution of blood (L/kg) x bodyweight (kg) x [desired PCV (%) – recipient PCV (%)] / donor PCV (%)*.

According to the calculations by Luethy et al. (2017), a volume of distribution of blood (VDB) of 0.103 l/kg should be assumed for alpacas, but the usually used VDB of 0.08 l/kg might also be suitable for a blood transfusion in alpacas.

A practical solution is a blood transfusion bag in which the donor animal’s blood is collected, mixed with anticoagulants, and then administered intravenously to the anaemic animal (Credille and Epstein 2016; Wittek and Franz 2021). Blood transfusion should always be preceded by crossmatching of the donor and recipient to minimise the risk of transfusion reaction (Credille and Epstein 2016). To date, only little information is available on the blood groups of SACs (Penedo et al. 1988), but according to Fowler and Bravo (2010), transfusion blood between different camelid species seems possible.

Luethy et al. (2017) evaluated the findings of 22 alpacas that received a total of 26 blood transfusions for various causes. The animals were not pre-treated and no cross-matching was performed. Adverse effects were observed in four transfusions, including pulmonary oedema, hyperthermia and irregular cardiac rhythm (Luethy et al. 2017).

## Conclusion and outlook

Anaemia is a common condition in alpacas and llamas, but in many animals it is detected late, as the animals can also compensate for an extremely low PCV for a long time. Early identification of anaemic animals can be done by regular assessment of the FAMACHA© score. For haematological analysis of blood samples from SACs the species specifics should be considered, which can lead to mismeasurements in automated analysers, especially in severely anaemic animals.

For detecting anaemic animals in time, we recommend regular (every 4 to 6 weeks) palpatory examination of BCS and FAMACHA© score (Wagener et al. 2022; Wagener and Ganter 2020). Animals that have a BCS < 2 and/or an FAMACHA© score > 2 should undergo further anaemia diagnostics. This consists at least of a faecal egg count and determination of the haematocrit by centrifugation in a capillary (Wagener et al. 2018b). If anaemia (PCV < 0.25 l/l) is confirmed, further laboratory diagnostic tests and calculations, including RBC count, haemoglobin, MCV, MCH, MCHC should be performed. Furthermore, an evaluation of the blood smear should always be performed in anaemic alpacas and llamas, as the appearance of normoblasts, anisocytosis, polychromasia and Howell-Jolly bodies may indicate a regenerative character of the anaemia. Regenerative anaemia can be distinguished by the amount of reticulocytes. If necessary, normoblast correction of the leukocytes should be calculated. In case of fatal anaemia (PCV < 0.10 l/l), blood transfusion from a healthy donor is necessary.

## Data Availability

All relevant data are included in the manuscript.

## Abbreviations

BCS: Body Condition Score
EDTA: Ethylenediaminetetraacetic acid
IMHA: Immune-mediated haemolytic anaemia
MCH: Mean corpuscular haemoglobin
MCHC: Mean corpuscular haemoglobin concentration
MCV: Mean corpuscular volume
PCV: Packed cell volume
RBC: Red blood cell
RDW: Red cell distribution width
SAC: South American camelid
VDB: Volume of distribution of blood
WBC: White blood cell
